# Sperm quality and quantity evolve through different selective processes in the Phasianidae

**DOI:** 10.1038/s41598-019-55822-3

**Published:** 2019-12-17

**Authors:** Wen Bo Liao, Mao Jun Zhong, Stefan Lüpold

**Affiliations:** 1 Key Laboratory of Southwest China Wildlife Resources Conservation (Ministry of Education), China West Normal University, Nanchong, 637009 Sichuan China; 2 Key Laboratory of Artificial Propagation and Utilization in Anurans of Nanchong City, China West Normal University, Nanchong, Sichuan 637009 China; 3 Institute of Eco-adaptation in Amphibians and Reptiles, China West Normal University, Nanchong, 637009 Sichuan China; 4 Department of Evolutionary Biology and Environmental Studies, University of Zurich-Irchel, 8057 Zurich, Switzerland

**Keywords:** Behavioural ecology, Sexual selection

## Abstract

Sperm competition is often considered the primary selective force underlying the rapid and diversifying evolution of ejaculate traits. Yet, several recent studies have drawn attention to other forms of selection with the potential of exceeding the effects of sperm competition. Since ejaculates are complex, multivariate traits, it seems plausible that different ejaculate components vary in their responses to different selective pressures. Such information, however, is generally lacking as individual ejaculate traits tend to be studied in isolation. Here, we studied the macroevolutionary patterns of ejaculate volume, sperm number, sperm length and the proportion of viable normal sperm in response to varying levels of sperm competition, body size and the duration of female sperm storage in pheasants and allies (Phasianidae). Ejaculate volume, sperm number and sperm viability were all relatively higher in polygamous than in monogamous mating systems. However, whereas ejaculate volume additionally covaried with body size, sperm number instead increased with the female sperm-storage duration, in conjunction with a decrease in sperm length. Overall, our results revealed important details on how different forms of selection can jointly shape ejaculates as complex, composite traits.

## Introduction

The ability of females to store sperm in their reproductive tract between copulating and fertilizing eggs is taxonomically widespread^1^. Sperm storage not only separates mating from fertilization but can also extend the temporal overlap between ejaculates from different males within the female reproductive tract, thereby enhancing the opportunity for postcopulatory sexual selection^2–4^. Postcopulatory sexual selection, encompassing both the competition for fertilization between sperm from different males^4^ and female contributions to its outcome^5^, enhances selection on ejaculate quality, in that sperm not only need to survive and retain their fertilizing capacity until the eggs have been fertilized, but also need to be more successful than their rivals in the process^6–9^.

Theory predicts that increased levels of sperm competition should, in principle, select for both more and higher-quality sperm^10^, a prediction that finds broad empirical support^6–9^. Yet, with many studies examining the response of individual sperm traits to postcopulatory sexual selection, such as sperm velocity^11^, sperm viability^12^ and particularly sperm morphometry^13–19^, our understanding of the evolution of ejaculates as composite traits is still rudimentary. Ejaculates are complex multivariate traits, of which different properties (e.g., sperm number, size and function) are unlikely to evolve independently^9^. In fact, this non-independence has been documented in experimental work showing how (competitive) fertilization success is determined by the interaction of multiple ejaculate traits^20,21^, as well as in comparative studies that have revealed how sperm quantity and multiple sperm-quality traits covary positively with one another and with a proxy of sperm competition^22–24^.

Whereas increased levels of sperm competition are predicted to enhance male investment in ejaculates overall^25^, approaching the capacity of sperm production under physical and energetic constraints^26,27^ should, based on theory^28–30^, generate trade-offs between sperm size and number. Which of these two ejaculate traits is favoured over the other by selection is thought to depend at least in part on the density of sperm prior to fertilization^30^ (but see ref. ^31^ for additional hypotheses). Stronger selection on sperm size than on sperm number is predicted when sperm operate under dense conditions and actively interact with one another within a relatively small female reproductive tract^30,32^, which often results in sperm displacement from storage sites^21,33^. However, when sperm are more diluted and competitive fertilization success is largely proportional to the relative abundance of competing sperm (i.e., raffle), then selection on ejaculates should favour sperm quantity over sperm size^30,32^. Empirical evidence for these predictions comes from comparative studies of both internally^32,34^ and externally fertilizing taxa^35^. Of these studies, however, Liao *et al*.’s^35^ comparison of anurans and fishes also demonstrates how the effects of sperm competition, the oftentimes assumed main driver of ejaculate evolution, can be greatly exceeded by other forms of selection on sperm size and number, such as sperm limitation through gamete dispersal in the fishes, for example.

Another taxon, in which sperm evolution appears to be dominated by other forms of selection than sperm competition, is that of the pheasants and allies (Phasianidae). Despite considerable variation in mating systems^36^, with associated diversity in premating display traits and behaviors^36^ and postcopulatory investments^37,38^, Immler *et al*.^39^ found no evidence for a link between sperm length and relative testes size as a proxy of sperm competition. Instead, sperm length was inversely related with the duration of female sperm storage, which varies between a few days and several weeks in this taxon^39^. Since sperm morphometry measures but one axis of ejaculate quality, however, this result does not negate any involvement of postcopulatory sexual selection in the evolution of ejaculates more broadly. Rather, the Phasianidae provide an opportunity to examine how ejaculates as multivariate traits respond to different, possibly interacting selection pressures, thereby potentially revealing constraints or functional links between different ejaculate parameters. For example, Immler *et al*.^39^ attributed the decline in sperm length with increasing sperm-storage duration to a possible trade-off between sperm length and sperm viability or longevity, a hypothesis that awaits formal testing. In principle, it is also possible that, after accounting for sperm competition, males of species with extended oviposition periods transfer relatively more sperm to ensure that even the last egg is reached and penetrated by sufficient sperm. This may be particularly important given that avian ova typically require polyspermic penetration for successful embryo development even though their pronucleus ultimately fuses with only a single sperm^40^. On their way to the egg, avian sperm are also stored for varying periods of time in tubular invaginations at the uterovaginal junction of the oviduct (i.e., sperm-storage tubules, SSTs) and are released at a species-specific, constant rate throughout the oviposition period^41–43^. The number of the SSTs scales with female body size^44^. Hence, in addition to the period of sperm storage and the associated passive sperm loss, differences in female body size, along with the abundance of SSTs and any dilution effects within the female reproductive tract, might also contribute to variation in ejaculate size across species. Finally, since only sperm that are viable, motile as well as morphologically normal can successfully negotiate the physically and biochemically challenging environment of the vagina and reach the SSTs^45^, males should not only transfer many, but many functional sperm. It is conceivable that maximizing the number of sperm entering and surviving in SSTs would become particularly important when prolonged sperm storage is combined with high levels of sperm competition. Thus, even if sperm morphometry was affected only by the female sperm-storage duration as reported^39^, both sperm quantity and quality could, additionally or instead, respond to sperm competition and other predictors.

Here, using literature-based data from 32 species of pheasants and allies in a phylogenetic framework, we aimed to disentangle the relative importance of sperm competition, allometric effects and female sperm storage in the (co)evolution of different ejaculate traits. Whilst the response of sperm morphometry to these selection pressures has been studied previously^39^, we focused on the ejaculate volume, total sperm number and the proportion of viable, morphologically normal sperm (henceforth ‘proportion of viable normal sperm’) in ejaculates, as well as their relationships with sperm morphometry, to better understand the evolution of ejaculates as composite traits. Within the proportion of viable normal sperm, spermatozoa with bent necks, broken tails, acrosome abnormalities or malformed heads were counted as abnormal sperm. We predicted that, even though sperm length itself may respond solely to the female sperm-storage duration^39^, both sperm quantity and functional quality should also increase with the level of sperm competition as outlined above. Finally, given the decrease in sperm length with increasing sperm-storage duration, we tested whether sperm length was indeed traded off against sperm viability as hypothesized by Immler *et al*.^39^, against sperm number, or both.

## Results

In a phylogenetic generalized linear model (PGLM)^46^ across all 32 species in our dataset, with phylogenetic relationships derived from Stein *et al*.’s^47^ time-calibrated, multi-gene phylogeny of Galliformes, the duration of female sperm storage was not affected by female body mass (*r* = 0.14 (95% confidence interval: -0.21 to 0.45), *t*_30_ = 0.78, *P* = 0.44) nor by the social mating system (monogamy/rare polygamy vs. obligate polygamy: *r* = 0.16 (-0.19 to 0.46), *t*_30_ = 0.90, *P* = 0.37; phylogenetic scaling factor λ = 0.97^<0.001,0.34^). The same was true when using relative testes mass as an index of sperm competition (body mass: *r* = 0.21 (-0.17 to 0.51), *t*_27_ = 1.11, *P* = 0.28; testes mass: *r* = 0.08 (-0.29 to 0.41), *t*_27_ = 0.39, *P* = 0.70). Unexpectedly, testes mass was not associated with the social mating system (*r* = -0.17 (-0.48 to 0.20), *t*_27_ = -0.91, *P* = 0.37) or body mass (*r* = 0.32 (-0.05 to 0.58), *t*_27_ = 1.73, *P* = 0.09; λ = 0.95^0.005,<0.001^). At least the association with body size might be weak because of six species with very small testes for their body size, including five with a polygamous mating system (Supplementary Fig. S1). We thus focus primarily on the social mating system in presenting our results below and provide in Supplementary Table S1 the corresponding results with relative testes mass as our proxy of sperm competition. Many results were qualitatively similar between the two indices.

### Relationships between ejaculate traits and breeding conditions

We tested for effects of sperm competition (social mating system or relative testes mass) and the duration of female sperm storage (total egg-laying period) on ejaculate traits. Except for sperm length, which Immler *et al*.^39^ reported to be highly repeatable (*R* = 0.94), we accounted for intraspecific variation in all ejaculate traits (see further details in *Material and Methods*). Throughout our analyses, we also tested for correlations among predictor variables and found no evidence for strong collinearity (all variance inflation factors, VIF ≤ 1.38).

**Figure 1:**
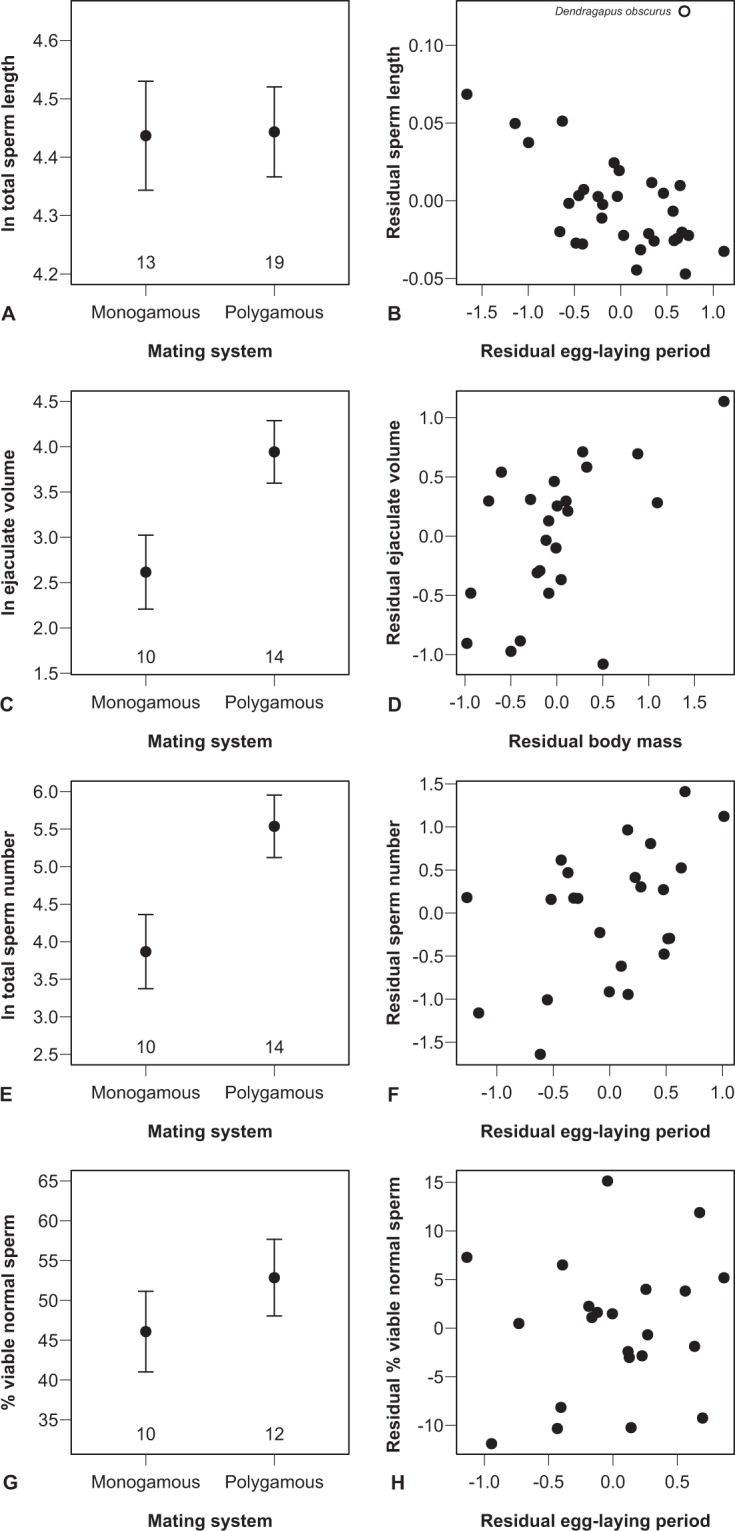
Graphical representation of the results of Table 1, showing the response of four ejaculate traits to (*left*) the social mating system as a proxy of sperm competition and (*right*) either the female egg-laying period or body mass. The left-hand panels depict the least-squares means with 95% confidence intervals, after controlling for body mass and the female egg-laying period (numbers at the bottom of each panel indicate sample sizes). The right-hand panels show the partial regressions derived from the same models, controlling for body mass and the social mating system on both axes. The labelled data point in panel B indicates *Dendragapus obscurus*, the removal of which considerably strengthened the negative relationship (see text and Table 1). The proportion of viable normal sperm was arcsine-square-root transformed and then converted to percentages by multiplying by 180/π. Total sperm length was measured in μm, ejaculate volume in μl, and total sperm number in millions.

Consistent with previous reports^39^, a PGLM across 32 species revealed that sperm length was unaffected by the social mating system and body mass (both |*r|* ≤ 0.22, |*t|* ≤ 1.12, *P* ≥ 0.24), but decreased with any increase in the egg-laying period (*r* = -0.38 (-0.62 to –0.02), *t* = -2.19, *P* = 0.04; λ < 0.001^1.00,<0.001^). Here, the dusky grouse *Dendragapus obscurus* was a very influential data point (Fig. 1), primarily due to its very long sperm (log scale: 5.08 vs. the 95% confidence interval around the mean of all species: 4.12–4.81). Removing this species considerably strengthened the negative effect of the egg-laying period, with no qualitative changes for the other predictors (Table 1 and Supplementary Table S1).

**Table 1:** Results of phylogenetically informed generalized least-squares models examining the effects of mating system, female egg-laying period (proxy of sperm-storage duration) and body mass on different ejaculate traits.

Response	Predictors	*r*	95% CL	*t*	*P*	*λ*
Total sperm length	Mating system	−0.10	−0.43, 0.27	−0.53	0.602	<0.001^1.00, <0.001^
(*N* = 31)*	**Egg-laying period**	**−0.69**	**−0.82, -0.45**	**−4.98**	**<0.001**	
	Body mass	−0.15	−0.47, 0.22	−0.80	0.431	
Ejaculate volume	**Mating system**	**0.72**	**0.44, 0.85**	**4.68**	**<0.001**	<0.001^1.00, 0.01^
(*N* = 24)	Egg-laying period	0.18	−0.25, 0.53	0.83	0.416	
	**Body mass**	**0.46**	**0.04, 0.70**	**2.31**	**0.032**	
Total sperm number(*N* = 24)	**Mating system**	**0.73**	**0.46, 0.85**	**4.79**	**<0.001**	<0.001^1.00, 0.01^
	**Egg-laying period**	**0.45**	**0.04, 0.69**	**2.27**	**0.035**	
	Body mass	0.07	−0.35, 0.45	0.65	0.752	
Proportion of viable normal sperm	**Mating system**	**0.49**	**0.06, 0.72**	**2.37**	**0.029**	<0.001^1.00, 0.002^
	Egg-laying period	0.14	−0.31, 0.52	0.61	0.550	
(*N* = 22)	Body mass	−0.18	−0.54, 0.28	−0.76	0.459	

All analyses except that on sperm length were weighted by 1/√SE, where SE refers to the intraspecific standard errors of the response variable. Statistically significant results are highlighted in bold.

^*^After exclusion of *Dendragapus obscurus* as an extreme outlier (Fig. 1).

**Figure 2:**
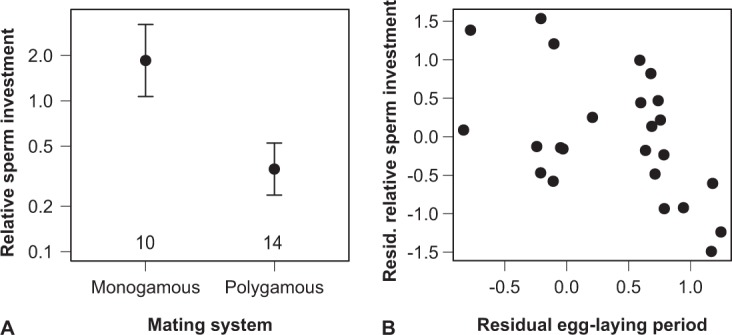
Graphical representation of the response of the relative sperm investment, ln(sperm length/sperm number), to (**A**) the social mating system as a proxy of sperm competition and (**B**) the female egg-laying period. (**A**) depicts the least-squares means with 95% confidence intervals and sample sizes, after controlling for the female egg-laying period and body mass. (**B**) shows the partial regression derived from the same model, controlling for body mass and the social mating system on both axes.

The ejaculate volume, the total number of sperm contained in it, as well as the proportion of viable normal sperm were all significantly larger in polygamous than in mostly monogamous species (Table 1). However, whilst the ejaculate volume additionally increased with body size, sperm number did so with the duration of female egg laying instead (Table 1). Using relative testes mass confirmed the effect of the sperm-competition index, but not the other effects (Supplementary Table S1). Since sperm length decreased, but sperm number increased with the female sperm-storage duration, we tested whether shorter sperm enhanced the response of sperm number to sperm storage. We used the ratio of ln(sperm length/sperm number) in ejaculates similar to theoretical^30,32^ and empirical work^32,34,35^ on the relative response of both ejaculate traits to selection. Although these aforementioned studies focused on sperm competition in different fertilization environments, the predictions are the same as in our case, namely that stronger selection on sperm number than sperm length should result in a negative response of this ratio. Controlling for intraspecific variation in both ejaculate traits, the relative gamete investment decreased with the sperm-storage duration (*r* = −0.54 (−0.74 to −0.15), *t*_20_ = −2.85, *P* = 0.01), in addition to being lower for polygamous species (*r* = −0.73 (−0.85 to −0.46), *t*_20_ = −4.79, *P* = 0.0001; body mass: *r* = −0.07 (-0.45 to 0.35), *t*_20_ = −0.30, *P* = 0.77; λ < 0.001^1.00,0.03^; Fig. 2). These results suggest that selection favours sperm number over sperm size, and the effect of sperm storage tended to be somewhat stronger when incorporating sperm length than for sperm number alone (Table 1).

### Correlations between ejaculate traits

**Figure 3:**
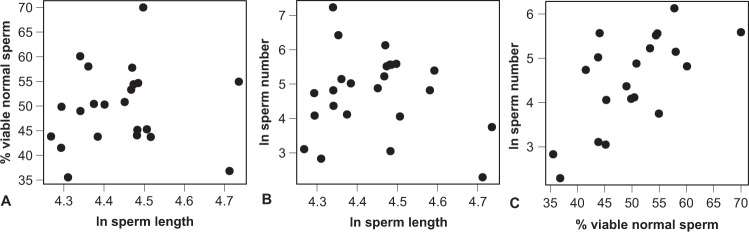
Pairwise correlations between three ejaculate traits. The proportion of viable normal sperm was arcsine-square-root transformed and then converted to percentages by multiplying by 180/π. Total sperm length and total sperm number were quantified in μm and millions, respectively.

To explore potential (co)evolution of different ejaculate traits, we examined the phylogenetically informed correlations between sperm length, the total number of sperm per ejaculate, and the proportion of viable, morphologically normal sperm, while again accounting for intraspecific variation. We found no significant association of sperm length with the proportion of viable normal sperm (*r* = 0.27 (−0.16 to 0.59), *t*_20_ = 1.27, *P* = 0.22; Fig. 3A), and this result did not change when using absolute or relative midpiece or flagellum lengths (all |*r*| < 0.30, *P* > 0.19), the two sperm components that have previously been shown to explain sperm function across species^19,48^. Sperm length also was not correlated with the total number of sperm per ejaculate (*r* = −0.01 (−0.39 to 0.38), *t*_22_ = −0.05, *P* = 0.96; Fig. 3B). By contrast, sperm number was strongly correlated with the proportion of viable normal sperm (*r* = 0.66 (0.30 to 0.81), *t*_18_ = 3.68, *P* = 0.002; Fig. 3C).

## Discussion

Across our set of phasianid species, we found the number of sperm per ejaculate and the proportion of viable normal sperm to covary positively with one another, to exhibit higher values in polygamous than in mostly monogamous species and, at least for sperm number, to increase with the duration of female sperm storage. By contrast, sperm length was not associated with other ejaculate traits and, confirming previous results^39^ in an expanded dataset, also covaried only with the female sperm-storage duration but not with sperm competition or body size.

Clearly, our analyses were conducted on a limited sample of species, considering that 187 different phasianid species are currently recognized worldwide^49^. Although many of the results reported here were relatively clear, it is too early for broad generalizations to all Phasianidae or even Galliformes. Yet, our study combines and expands on two previous reports, one on the evolution of sperm morphometry^39^ and the other on multiple ejaculate traits in a non-evolutionary context^50^, and yields novel insight into possible selection pressures on, and (co)evolution of, ejaculate traits. Importantly, our results highlight that ejaculate traits are differentially linked with one another and respond to different selection pressures. Whereas sperm competition probably is the most widely studied, and often the only evolutionary force considered, to explain the diversification of ejaculate traits, our analyses show that the duration of female sperm storage also explains a significant portion of the variation in both sperm length and number. Hence, joint examination of interconnected traits and their responses to both natural and sexual selection can yield a broader and more complete picture of the evolutionary processes driving the evolution of complex traits such as ejaculates. We hope that such an approach will also be used in future research.

Consistent with sperm competition theory^28,51^, we found polygamous species to invest significantly more in their ejaculates than their (mostly) monogamous counterparts, in terms of both semen volume and gametic content. The ejaculate volume additionally increased with body size, possibly through size-related investments by males or in response to potential dilution effects in the female reproductive tract. Interestingly, the number of sperm was independent of body size even though body size is associated with the number of sperm-storage tubules that these sperm could occupy^44^. Instead, sperm number increased with the duration of female sperm storage. Since prolonged sperm storage was also associated with shorter sperm^39^, this result suggests that sperm numbers might be under particularly strong selection through the combination of sperm competition and sperm storage patterns. In fact, analysing both sperm length and number combined indicated that shorter sperm might indeed enhance the increase in sperm numbers in response to the female sperm-storage duration. This decrease in sperm length has previously been hypothesized to result from a trade-off between sperm length and longevity^39^. We did not find evidence for such a trade-off, at least to the extent that the proportion of viable normal sperm is correlated with sperm longevity (see below). Rather, our findings suggest an additional or alternative explanation, namely that this decrease could be a response to an increased sperm demand to ensure that enough sperm reach the last egg to be fertilized despite the continuous sperm loss from storage. More direct examination of sperm storage in future research might provide a clearer picture of these different processes.

Sperm quantity was also positively correlated with the proportion of viable normal sperm, thus adding another example of possible coevolution between different ejaculate quality traits in response to sperm competition^22,23^. It is now well established that postcopulatory sexual selection should not only favour greater sperm quantities but also superior sperm quality^6–8^. In internal fertilizers, the female reproductive tract tends to be highly selective and so to exclude the vast majority of transferred sperm from ever reaching the site of fertilization^52,53^. In birds, many sperm may not even reach the SSTs^53,54^. Hence, there should be strong selection on maximizing the number of sperm transferred that are capable of negotiating these challenges, even more so in polygamous species due to added selection pressure through sperm competition^32,34^.

The proportion of viable normal sperm did not correlate with the duration of sperm storage. Although sperm need to be viable and morphologically and functionally normal to reach the infundibulum and successfully fertilize an egg, the proportion of viable normal sperm as a snapshot of sperm quality at the time of sperm release does not directly measure the functional lifespan of sperm, which is largely driven by the rate of viability loss over time. Even though it appears rather unlikely that species in which males transfer ejaculates with many unviable sperm are under strong selection to produce long-lived sperm, species with high initial sperm viability could still differ dramatically in sperm longevity, depending on the period over which eggs are laid and sperm need to maintain their fertilizing capacity. Our results suggest a possible indirect association between sperm viability and the duration of sperm storage, with males of species with extended sperm storage releasing more sperm, which in turn is positively associated with the proportion of viable normal sperm. If so, a higher proportion of functional sperm might primarily increase the number of sperm populating the numerous SSTs to ultimately ensure that sufficient sperm reach the last egg of a clutch despite constant passive sperm release from these tubules^41–43^. Once sperm are inside an SST, their motility is thought to be suppressed and their survival to be maintained at least in part by secretions of the sperm-storage tubules^55^. However, it has also been postulated that in the domestic fowl *Gallus gallus domesticus*, sperm metabolism, which depends on mitochondrial (i.e., midpiece) function, is an important contributor to the duration of sperm storage^56^. It thus remains unclear just how metabolically active or quiescent sperm are within the storage tubules, and thus if and how exactly sperm viability and longevity are connected. Further research on this topic might provide an answer.

That we found only somewhat overlapping results between our different proxies of sperm competition (i.e., social mating system vs. relative testes mass) could be for at least four reasons. First, phasianid genetic mating systems, and particularly their levels of mixed paternity, remain elusive but are thought to be highly diverse^36,57^. We reduced the three categories of Immler *et al*.’s dataset^39^ to two due to our small sample size for some ejaculate traits and because none of our initial analyses revealed a difference between monogamous and rarely or facultatively polygamous species. Our main results did not change qualitatively when using three groups, but naturally any potential misclassification of mating systems can affect their prediction of ejaculate traits. Second, due to dramatic seasonal variation in the testes of birds, any deviation from the peak of the season can greatly underestimate their true size^58^. Although all testes seem to have been measured in reproductively active males, the different sources did not specify when exactly testes were measured relative to the seasonal peak, leaving the possibility that some testes might not have been fully developed (e.g., see Supplementary Fig. S1). Third, in addition to manufacturing sperm, testes also produce testosterone, which is critical for many premating sexual traits and behaviours in pheasants^59,60^. Thus, variation in both the proportion of sperm-producing tissue within the testes^26,27^ and its efficiency in producing sperm^61,62^ might somewhat obscure the interspecific relationship between relative testes size and the level of sperm competition. Finally, relatively large testes can be an adaptation to both sperm competition and frequent copulation^63^, such that a polygynous species might have relatively low levels of sperm competition if males can monopolize access to females^37^, but still have large testes in response to a high mating frequency^63^. Without further information it is difficult to judge why relative testes mass was not significantly correlated with the social mating system. Overall, however, our results with the social mating system seem to align more closely with theoretical predictions than relative testes size. Whether this is true indeed will need further examination.

In conclusion, we here expanded on earlier reports on the evolution of sperm morphometry in the pheasants and their allies^39^, by including further species and additional ejaculate traits to paint a more nuanced picture of the evolutionary processes underlying the evolution of ejaculates as complex, composite traits. Our results suggest separate, but interconnected selection pressures on different ejaculate traits, including possible indirect selection and evolutionary constraints or trade-offs. These findings add another example to a short but growing list of studies providing comparative evidence for the interplay between different selective processes in the diversification of ejaculates as well as the multivariate evolution of ejaculates themselves.

## Material and Methods

### Data collection

We compiled data from the literature on the social mating system, body mass, combined testes mass, different ejaculate traits (ejaculate volume, sperm length, sperm number and the proportion of viable and morphologically normal sperm), and the duration of female oviposition for 32 phasianid species non-perdicine (Supplementary Data), covering approximately 17% of all 187 extant species in this family^49^. We omitted the specious subfamily of the Perdicinae (*N* = 111), which is paraphyletic with respect to the remaining Phasianidae (Phasianinae, Meleagridinae, Tetraoninae), because it was represented by only 2–4 species for the different ejaculate traits despite extensive data search, thus introducing a phylogenetic bias. Further, at least quails (*Coturnix* spp.) in this taxon differ from the other phasianid species by producing both unusually long sperm^64^ and during copulation transferring their semen along with a foamy cloacal fluid that affects sperm function^65,66^. For these reasons, we restricted our data to the 32 non-perdicine species for consistency. Further, although *Lophura edwardsi* and *L. hatinhensis* were treated as a separate species in our data sources, they are now both considered one species^67^. We thus used only the data identified as *L. edwardsi* as in Stein *et al*.’s^47^ phylogeny. Their representation among all 76 non-perdicine species did not deviate from a random distribution (likelihood-ratio test: χ^2^ = 1.47, *P* = 0.22) and all traits examined showed considerable interspecific variation, thus indicating a representative and unbiased sample of this taxon.

Due to our relatively small dataset and no differences between Immler *et al*.’s^39^ mating-system scores of “polygamy not recorded” and “polygamy facultative or rare” in any of our analyses, we combined these two categories, thus distinguishing between (1) monogamy or rare/facultative polygamy and (2) frequent/obligate polygamy.

Data on sperm length, social mating system, body mass and female oviposition were available for all 32 species, those on ejaculate volume and total sperm numbers each for 24 species, whilst the proportion of viable normal sperm was recorded in only 22 species (Table S1). Complete data across all focal traits were available for 20 species. Nearly all data on ejaculate traits (*N* = 21–22 species) came from the same study, collected by the same team^50^ (with corresponding sperm morphometrics reported in ref. ^39^; see Supplementary Data), thus minimizing variation due to methodological differences between species. In all these species, semen samples were obtained by dorso-abdominal massage^68^, which is a widespread collection technique in large birds. The blue peafowl *Pavo cristatus* and the western capercaillie *Tetrao urogallus* were the only exceptions, with ejaculates collected using teaser females. Although these two techniques may yield slightly different sperm quantities (e.g., *Coturnix japonica*: 14.8 vs. 18.7 × 10^6^ sperm^69,70^; *T. urogallus*: 49.2 vs. 39.3 × 10^6^ sperm^71^), such deviations seem negligible compared to the over 100-fold greater variation across species. A more widespread source of intraspecific variance is general within- and between-male variation in ejaculate quantity and quality, combined with possible sampling variation, even though such variation is expected to introduce random error rather than a systematic bias across the phylogeny. To address this issue, we first used Saint Jalme *et al*.’s^50^ original dataset to calculate the intraspecific repeatability across 18 species with at least two samples measured (Supplementary Fig. S2), using the flexible mixed-model-based repeatability analysis in the R package *rptR*^72^. All traits were significantly repeatable (log ejaculate volume: *R* = 0.59 (95%CI = 0.36–0.71); log sperm number: *R* = 0.56 (0.32–0.72); proportion of viable normal sperm (binomial error distribution): *R* = 0.35 (0.13–0.61); note that Immler *et al*.’s^39^ repeatability for sperm length was *R* = 0.94, albeit for intra-ejaculate repeatability on a single sample per species). Second, we accounted for heterogeneity in intraspecific variance for all ejaculate traits except for sperm length as described in *Statistical analyses*. Our calculated trait-specific sample sizes and means from Saint Jalme *et al*.’s^50^ dataset occasionally deviated from the published values because not all measures were available for each sample and because we removed a few very influential outliers. Our data with species-specific standard errors and samples sizes for each trait are available in the Supplementary dataset.

We estimated the duration of female sperm storage as the period of laying one full clutch of eggs, i.e., the species-specific mean clutch size multiplied by the typical interval between consecutive eggs (minus one interval). Since many bird species copulate only before laying the first egg of a clutch^2,73^, this oviposition period is a good proxy of the species-specific sperm-storage duration^44^. Due to missing information, we omitted any variation in sperm storage up to the first egg (e.g., peahens can cease copulating 12 days before laying their first egg^73^), such that our estimates of sperm storage are relatively conservative. In our dataset, this egg-laying period ranged from one day in *Polyplectron germaini* (2 eggs one day apart) to 21 days in *Meleagris gallopavo* and *Phasianus colchicus* (average of 11.5 eggs at 2-day intervals).

### Statistical analyses

We performed all statistical analyses using R v.3.6.0 and log-transformed all continuous variables except for the proportion of viable normal sperm, which we transformed by the arcsine square root. To account for the statistical nonindependence of data due to shared ancestry, we performed phylogenetically informed analyses based on Stein *et al*.’s^47^ time-calibrated, multi-gene phylogeny of 225 extant Galliformes. Specifically, we conducted generalized least-squares (GLS) models in the R package *nlme*^74^, incorporating Pagel’s^75^ phylogenetic correlation structure (corPagel) as implemented in the *ape* package^76^ to estimate the phylogenetic scaling parameter λ using maximum likelihood. We then compared the fit of these models with that of models that had λ fixed to either 0 (phylogenetic independence) or 1 (complete phylogenetic association). The *P*-values of these comparisons are indicated by superscripts following each λ value. For phylogenetic product-moment correlations between ejaculate traits, we used a Brownian correlation structure (corBrownian)^76^.

To account for intraspecific variation in ejaculate volume, sperm numbers or the proportion of viable normal sperm, we weighted all GLS models involving these variables by the inverse of the square-root-transformed intraspecific standard errors^77^. Where two of these traits were included in the same analysis, these weights were combined using the *varComb* function. For species with only one sample measured or no standard error/deviation reported (1–3 species per trait), we imputed the intraspecific variance based on the pooled variances of all other species, weighted by their sample sizes^77,78^. Before these conversions, variances of log-transformed variables were brought to the same scale by a log$(1+{\sigma }_{i}^{2}/{\bar{x}}_{i}^{2})$ transformation, where ${\bar{x}}_{i}$ and *σ*_*i*_^2^ are the intra-specific mean and variance, respectively^77^.

## Supplementary information

 Supplementary material

DATASET 1
