# Supplementary material for: Sperm quality and quantity evolve through different selective processes in the Phasianidae
Source: Sci Rep. 2019 Dec 17;9:19278. doi: 10.1038/s41598-019-55822-3 (PMC6917726; doi:10.1038/s41598-019-55822-3)
Supplement: Supplementary file 1 — Supplementary material [file 41598_2019_55822_MOESM1_ESM.pdf]

Supplementary material to:

Sperm quality and quantity evolve through different selective processes in the Phasianidae

Wen Bo Liao[1,2,3*], Mao Jun Zhong[1,2,3], Stefan Lüpold[4]

[1]Key Laboratory of Southwest China Wildlife Resources Conservation (Ministry of Education), China West Normal University, Nanchong 637009, Sichuan, China

[2]Key Laboratory of Artificial Propagation and Utilization in Anurans of Nanchong City, China West Normal University, Nanchong, Sichuan, 637009, China

[3]Institute of Eco-adaptation in Amphibians and Reptiles, China West Normal University, Nanchong, 637009, Sichuan, China

[4]Department of Evolutionary Biology and Environmental Studies, University of Zurich-Irchel, 8057 Zurich, Switzerland

*E-mail: Liaobo_0_0@126.com

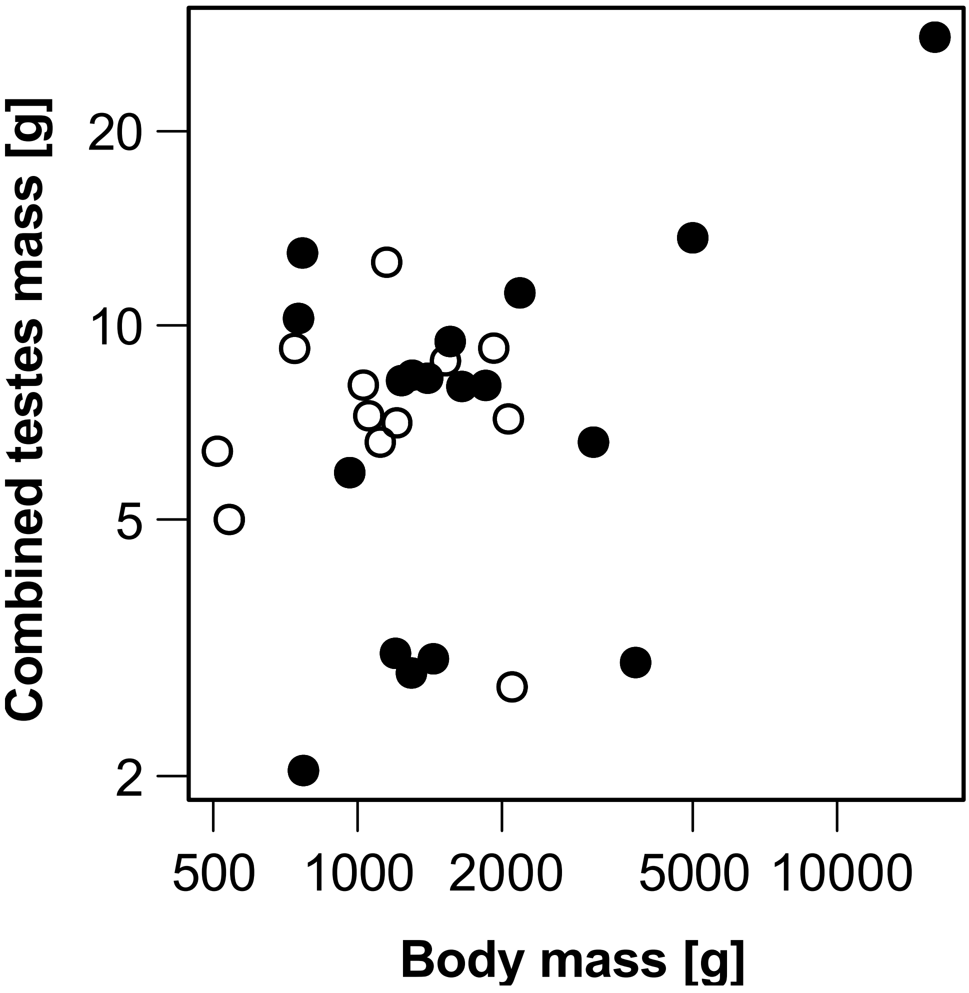

Supplementary Figure S1: Relationship between combined testes mass and body mass across 30 species of Phasianidae, separated by social mating systems (*open circles:* monogamous or rarely polygamous species; *filled circles:* polygamous species).

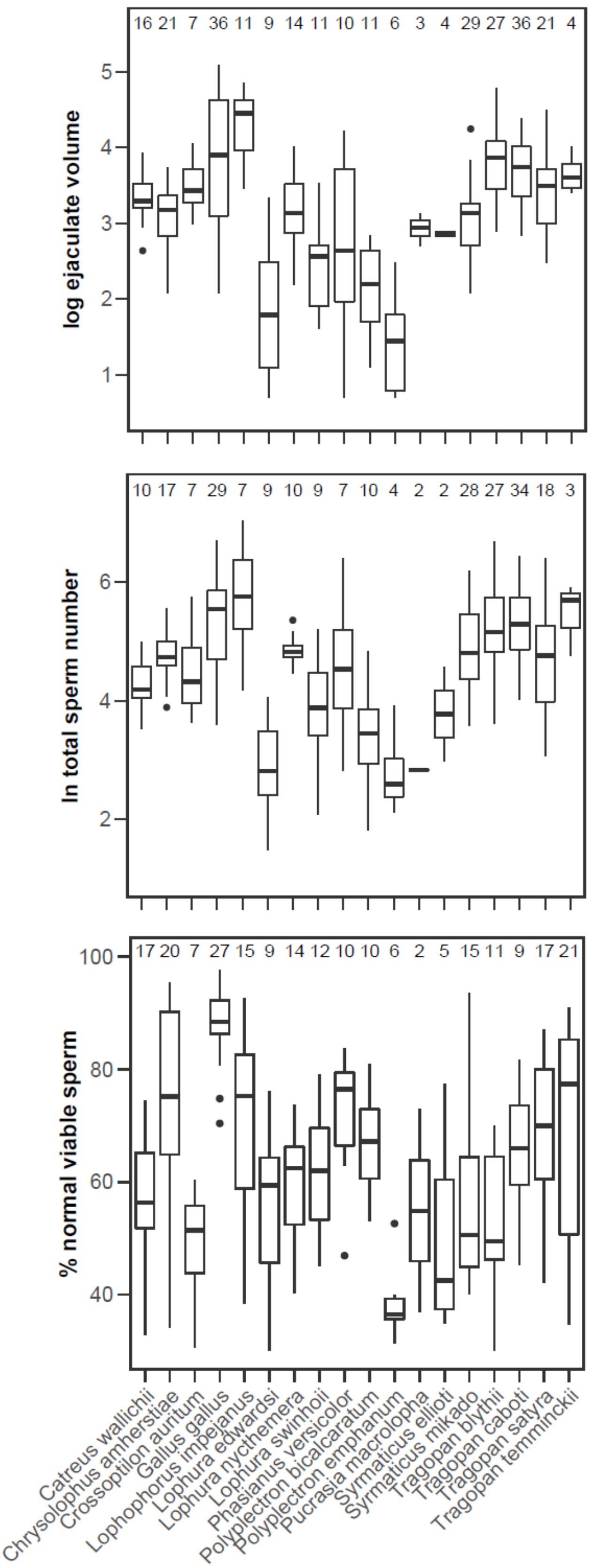

Supplementary Figure S2: Intraspecific variation in ejaculate traits, based on the sample-specific data from Saint Jalme et al.'s study (ref. 49 in main text). Numbers above boxes indicate sample sizes.

Supplementary Table S1: Results of phylogenetically informed generalized least-squares models examining the effects of testes mass, female egg-laying period (proxy of sperm-storage duration) and body mass on different ejaculate traits. All analyses except on sperm length were weighted by $1/\sqrt{SE_i}$, where SE_i represents the intraspecific standard errors of the response variable. Statistically results are highlighted in bold.

Response	Predictors	*r*	95% CL	*t*	*P*	*λ*
Ejaculate volume	**Testes mass**	**0.52**	**0.11, 0.74**	**2.65**	**0.016**	$0.71^{0.05,\ 0.18}$
(*N* = 23)	Egg-laying period	0.17	–0.27, 0.53	0.77	0.453	
	Body mass	0.35	–0.10, 0.64	1.62	0.121	
Total sperm number	**Testes mass**	**0.57**	**0.18, 0.76**	**2.99**	**0.008**	$0.66^{0.04,\ 0.03}$
(*N* = 23)	Egg-laying period	0.22	–0.22, 0.56	1.00	0.328	
	Body mass	0.11	–0.32, 0.49	0.48	0.636	
Total sperm length	Testes mass	–0.23	–0.53, 0.16	–1.20	0.243	$<0.001^{1.00,\ <0.001}$
(*N* = 30)	**Egg-laying period**	**–0.43**	**–0.66, –0.09**	**–2.40**	**0.024**	
	Body mass	–0.11	–0.45, 0.26	–0.58	0.568	
Total sperm length	Testes mass	0.03	–0.34, 0.39	0.13	0.895	$<0.001^{1.00,\ <0.001}$
(*N* = 29)*	**Egg-laying period**	**–0.72**	**–0.83, 0.48**	**–5.18**	**<0.001**	
	Body mass	–0.18	–0.50, 0.21	–0.93	0.363	
Proportion of viable	Testes mass	0.45	0.01, 0.71	2.07	0.054	$<0.001^{1.00,\ 0.002}$
normal sperm	Egg-laying period	0.00	–0.43, 0.43	0.00	0.999	
(*N* = 21)	Body mass	0.01	–0.42, 0.44	0.06	0.954	

*after exclusion of *Dendragapus obscurus* as an extreme outlier (cf. Fig. 1)
